# Case report: A novel transcutaneous electrical nerve stimulation improves dysesthesias and motor behaviors after transverse myelitis

**DOI:** 10.3389/fnhum.2024.1447029

**Published:** 2024-11-06

**Authors:** Yuki Nishi, Koki Ikuno, Yuji Minamikawa, Michihiro Osumi, Shu Morioka

**Affiliations:** ^1^Institute of Biomedical Sciences (Health Sciences), Nagasaki University, Nagasaki, Japan; ^2^Neurorehabilitation Research Center, Kio University, Nara, Japan; ^3^Department of Rehabilitation Medicine, Nishiyamato Rehabilitation Hospital, Nara, Japan

**Keywords:** transcutaneous electrical nerve stimulation, tingling, allodynia, upper limb activity, transverse myelitis, case report

## Abstract

**Purpose:**

Transverse myelitis (TM)-associated dysesthesia is diverse and frequently resistant to treatment. This study explored the comprehensive effects of a novel transcutaneous electrical nerve stimulation (TENS) approach tailored to an individual’s specific dysesthesia profile in a patient with TM.

**Patient and method:**

A 52-year-old woman with severe dysesthesias in the left C8 sensory area caused by TM underwent an A-B-A-B-A design intervention. The baseline (phase A) and intervention (phase B) phases were both 7 days. Tingling and allodynia were scored on an 11-point numerical rating scale pre-, post-, and 1 h post-treatment. Upper limb activities during daily living were assessed using a wrist-worn accelerometer. The intervention phase consisted of 60-min sessions of TENS two times daily. Furthermore, the intervention and carry-over effects of TENS were evaluated using Tau-U and Bayesian unknown change point models.

**Results:**

The effects of TENS resulted in the immediate improvement in tingling, allodynia, and upper limb activity. Long-term effects of TENS affected tingling and upper limb activity; however, no impacts on allodynia were observed.

**Conclusion:**

This novel TENS approach shows promise as an effective treatment, even in rare and treatment-resistant dysesthesia associated with TM.

## 1 Introduction

Transverse myelitis (TM) is a rare inflammatory neurological disorder affecting the spinal cord. With an estimated incidence rate of 3 cases/100,000 patient-years (Berman et al., 1981; Jeffery et al., 1993), the prognosis for TM recovery varies. Over 60% of patients experience persistent sequelae, and 44% experience mild-to-severe sequelae (Christensen et al., 1990; Ropper and Poskanzer, 1978). TM is characterized by motor, sensory, and autonomic impairments, along with pain below the lesion level (Frohman and Wingerchuk, 2010). Myelitis pain, with an unclear mechanism, is often refractory to treatment (Tackley et al., 2017; Zhao et al., 2014). Severe pain leads to motor behavior changes, prolonged physical impairments, and more significant disability (Asmundson et al., 1999; Leeuw et al., 2007).

Although pharmacological treatments are common for managing dysesthesia, systematic reviews show low effectiveness and a high risk of adverse events (Snedecor et al., 2013; Teasell et al., 2010). Transcutaneous electrical nerve stimulation (TENS) is a safe and inexpensive non-pharmacological treatment for neuropathic pain (Sluka, 2016). A novel TENS approach, dysesthesia-matched TENS (DM-TENS), tailors the stimulation parameters to match the individual’s specific dysesthesia profile (Nishi et al., 2022). DM-TENS has shown promise in improving allodynia, tingling, and mechanical hypoesthesia compared with conventional TENS, making it effective for difficult-to-treat dysesthesias in rehabilitation settings (Nishi et al., 2022). It also exhibits a unique phenomenon where the sensations of tingling and electrical stimulation appear to cancel each other, compared to conventional TENS.

Due to the difficulty in achieving an adequate sample size using traditional experimental methods for rare TM, reporting the characteristics and interventions conducted on an individual with TM is clinically significant. This study investigated the treatment effects of DM-TENS in a patient with TM who had chronic dysesthesias in the C8 sensory area. The comprehensive treatment effects of DM-TENS were verified using a longitudinal assessment of the dysesthesias and upper limb activities.

## 2 Case description

A 52-year-old female presented to our hospital with intractable severe dysesthesia in the left C8 sensory area associated with TM. She first noticed abnormal sensations in her left upper limb 3 years ago. Magnetic resonance imaging (MRI) revealed a high T2 signal extending from the C4 to Th2 spinal cord segments, with particularly intense T2 signals at the C6/7 level (Supplementary Figure S1A,C). The patient underwent extended laminectomy. Results of cerebrospinal fluid cytology, markers for malignant lymphoma, and anti-aquaporin-4 antibodies were all negative. The peripheral nerve conduction velocity of the ulnar nerve was normal. Histopathological examination revealed inflammatory cell infiltration. Considering these findings, myelitis was suspected, and steroid pulse therapy was administered. Consequently, the high signal on T2-weighted images decreased, except for that in the left lateral funiculus at C6/7 (Supplementary Figures 1B,D). Sensory and motor impairment were observed below the C8 and C7 levels, respectively. While the right upper limb had normal deep tendon reflexes (DTRs), the left upper limb had hyperactive DTRs. Supplementary Table 1 presents the DTR, manual muscle testing (MMT), and sensory examination details. No notable change in dysesthesias was observed, and TM did not worsen on periodic MRI. Standard neuropathic medications (5 and 60 mg of prednisolone and duloxetine, respectively, administered once daily, 300 mg of pregabalin administered two times daily, 112.5 mg of tramadol hydrochloride, and 975 mg of acetaminophen administered three times daily) were prescribed. She exhibited spontaneous tingling and allodynia in the left C8 sensory area, along with an abnormal squeezing-like sensation localized to the trunk and electric shock pain in the left lower limb. Specifically, the symptoms in the left C8 sensory area caused distress and fear, leading her to always wear a glove on her left hand and avoid its use. Because her job required typing on a computer, she was distressed by her inability to place her left forearm on the table and type using her left hand. She underwent outpatient physiotherapy two times weekly for 6 months at our hospital.

The Institutional Ethics Board of the Nishiyamato Rehabilitation Hospital approved this study. We explained the study protocol to the patient and obtained written informed consent to publish the case report and Supplementary Video 1.

## 3 Intervention

### 3.1 Intervention protocol

**Figure 1:**
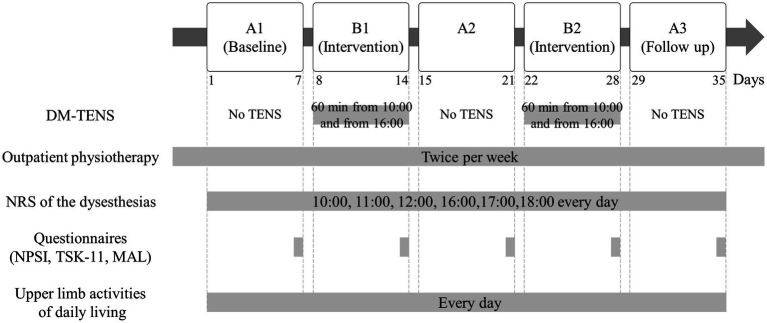
Flow diagram of the interventional procedures. Dysesthesia-matched transcutaneous electrical nerve stimulation (DM-TENS) was performed in phases B1 and B2.

Figure 1 presents the interventional design and evaluation. The intervention used an A-B-A-B-A design, with phases A and B as the baseline without TENS and DM-TENS intervention, respectively. All phases lasted 1 week, and the patient underwent outpatient physiotherapy two times weekly. Phase A1 was the pre-intervention phase. DM-TENS intervention was performed in phases B1 and B2. Phase A2 served as the post- (phase B1) and pre- (phase B2) DM-TENS phases to evaluate the stability or regression of the pre-intervention phase (phase A1) during the non-intervention period. Phase A3 included the post-DM-TENS (phase B2) and follow-up phases. Our previous study involved a case series comparing conventional high-frequency TENS with DM-TENS across multiple patients with spinal cord dysfunction (Nishi et al., 2022). The focus was on immediate effects. Here, the A-B-A-B-A design was selected to maximize our findings’ robustness. By alternating between no TENS (phase A) and DM-TENS (phase B), we aimed to minimize confounding effects and attribute any observed changes to the DM-TENS approach. This design allows for a detailed assessment of the immediate and carry-over effects of DM-TENS, providing a more in-depth evaluation over time.

### 3.2 Therapeutic intervention

#### 3.2.1 DM-TENS

Electrical stimulation (Espurge, Ito Physiotherapy, and Rehabilitation Co., Japan) was conducted for DM-TENS using a continuous pulse pattern, 50-μsec pulse duration, and a biphasic current with a symmetrical waveform. Self-adhesive 5 × 5 cm electrodes (Axelgaard Manufacturing, United States) were attached to the wrist over the left C8 sensory area, innervating the dysesthesia area (Supplementary Figure 2). The distance between the TENS electrodes’ center was 6 cm. The DM-TENS parameters were set at a frequency matching the spontaneous tingling beats and a stimulus intensity matching to the spontaneous dysesthesia intensity. The patient received instructions on using DM-TENS and adjusting the settings according to the abovementioned procedure; subsequently, she performed DM-TENS independently at home. DM-TENS was performed for 60 min two times daily at approximately 10:00 and 16:00 h during the DM-TENS intervention phase (B1 and B2). On workdays, she performed the intervention at 10:00 h while working. DM-TENS was set to terminate automatically after 60 min. The DM-TENS parameters were consistently set to 70 Hz frequency and 24–29 mA electrical intensity. At 50 μsec and 70 Hz, the patient’s sensory threshold was approximately 20 mA.

#### 3.2.2 Outpatient physiotherapy

The patient underwent outpatient physiotherapy twice per week during all phases, comprising 60 min of stretching, aerobic exercise, and progressive resistance training. At home, she was instructed to perform individualized self-exercise, including stretching and gait training. She was also educated on pain management and was advised to use her left hand following the dysesthesia condition rather than excessively immobilizing it.

#### 3.2.3 Dysesthesia assessment

Since the patient had tingling and allodynia in the left C8 sensory area, which interfered with her quality of life, the tingling degree and allodynia were selected for assessment and rated on an 11-point numerical rating scale (NRS) referring to the items of the self-administered neuropathic pain symptom inventory (NPSI). The NRS for assessing allodynia and tingling was self-administered at pre-treatment (10:00 and 16:00 h), post-treatment (11:00 and 17:00 h), and 1 h post-treatment (12:00 and 18:00 h) daily, and the scores for each time were averaged. All self-administered NPSI items were assessed on the last day of each phase.

#### 3.2.4 Kinesiophobia assessment

Since the patient had an excessive fear of using the affected hand, kinesiophobia was assessed employing the 11-item version of the Tampa Scale for Kinesiophobia (TSK) in Japanese on the last day of each phase (Woby et al., 2005).

#### 3.2.5 Upper limb activities of daily living

Upper limb activities of daily living (ADL) were measured subjectively and objectively. Subjective measurement of upper limb use was assessed using the motor activity log (MAL) on the last day of each phase (van der Lee et al., 2004). Objective measurement of upper limb use was performed using bilateral wrist-worn accelerometers (AX3, Axivity, United Kingdom). The patient was instructed to wear the accelerometers for at least 8 h daily for ≥4 days during each phase (1 week). We calculated the variables of upper limb activity with reference to previous studies (Bailey et al., 2015; Waddell et al., 2017). The bilateral magnitude was calculated per sample by summing the vector magnitude of both accelerometers. Additionally, the magnitude ratio was calculated as a natural logarithm transformation per sample by dividing the vector magnitude of the painful left upper limb by that of the non-painful right upper limb. Positive values indicated that non-painful upper limb activity was more than painful upper limb activity, whereas negative values indicated the opposite. The median bilateral magnitude and magnitude ratio were calculated as the variables representing upper limb activity daily.

#### 3.2.6 Statistical analyses

Dysesthesia variables were the pre-treatment, post-treatment, and 1-h post-treatment NRS scores of tingling and allodynia. Upper limb activity parameters were the median magnitude ratio and bilateral magnitude.

The effect size for DM-TENS was calculated using Tau-U analysis of dysesthesias and upper limb activities (Brossart et al., 2014; Parker et al., 2011). Tau-U calculations were performed using a web-based calculator^1^ (Vannest et al., 2016). We corrected the baseline when values exceeded 0.20; the trend was characterized in the same direction as the intervention aim. Following the guidelines, Tau-U values <0.2, 0.2–0.6, 0.6–0.8, and > 0.8 were considered small, medium, large, and very large, respectively (Vannest and Ninci, 2015). Statistical significance was set at *p* < 0.05.

Bayesian unknown change point (BUCP) models can investigate and quantify the presence of immediate or carry-over effects by detecting abrupt changes in observations across the phases (Natesan Batley et al., 2020a,b; Natesan and Hedges, 2017). For each time series of dysesthesia and upper limb activity parameters, the boundary between the phases was considered unknown, and priori distribution was set at a uniform distribution. The marginal posterior probabilities over the combinations of the change-point locations and probabilities for each change-point were calculated using Bayesian inference. If the correct combination of change points has the maximum probability, evidence for immediacy was deemed sufficient.

## 4 Results

Adherence to the treatment procedures was confirmed. The patient reported diminished sensations of dysesthesias and electrical stimulation with DM-TENS use, and easier hand movement. She generally did not require adjustments to the stimulation intensity due to habituation, as they did not report any decrease in sensation or therapeutic effect during the 60-min sessions. However, on the second day of phase B1, a minor adjustment was made during DM-TENS treatment, increasing the intensity from 25 to 28 mA due to a slight increase in the dysesthesia. No adverse effects were reported. On workdays and during treatment phases, typing was performed while using DM-TENS. As the patient could place her left forearm on the table and type using her left hand, she felt relieved from the stress she experienced. On days 12–14 and 24–28 of phases B1 and B2, respectively, the patient did not receive tramadol at 13:00 h. From days 24–28 of phase B2, she removed the glove on her left hand but wore it again during phase A3. Supplementary Table 2 presents NPSI items.

### 4.1 Effect of DM-TENS on tingling

**Figure 2:**
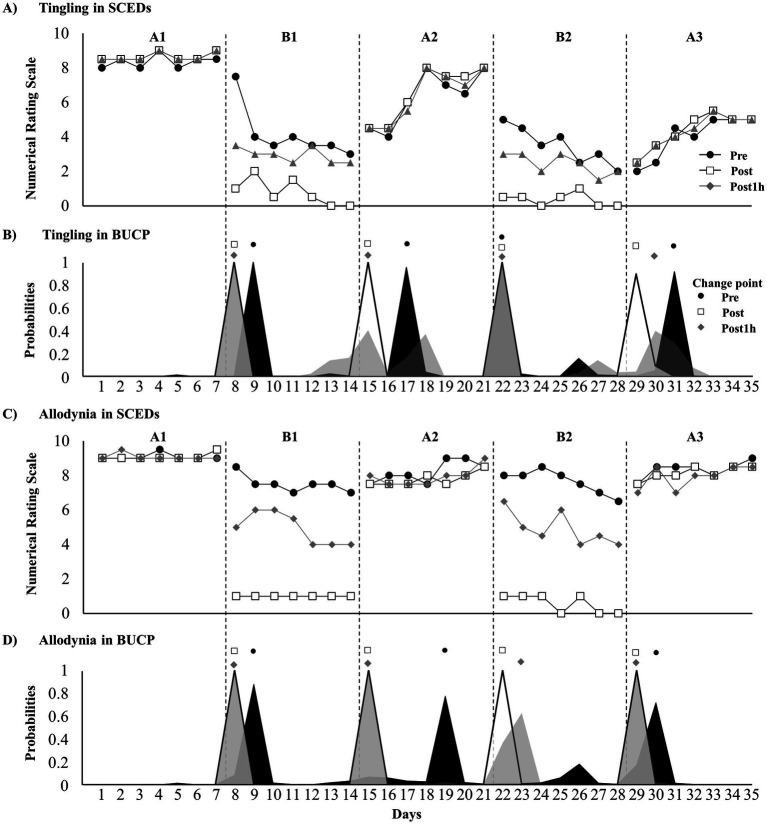
Time course of dysesthesia and change point probabilities. **(A)** Time course of tingling; **(B)** probabilities of tingling change points; **(C)** time course of allodynia; and **(D)** probabilities of allodynia change points.

Figure 2A presents the tingling at pre-treatment, post-treatment, and 1-h post-treatment. Tau-U analysis revealed significantly improved treatment effects at pre-treatment, post-treatment, and 1-h post-treatment (Table 1). The BUCP model identified four pre-treatment change points with a 77.1% probability, estimated to be on days 9, 17, 22, and 31, with a 97–100% probability of being accurately estimated. Four change points were found for post-treatment with a 99.7% probability, estimated to be on days 8, 15, 22, and 29, with a 99.6–100% probability of being estimated accurately. Furthermore, four pre-treatment change points had a 65.8% probability, estimated to be on days 8, 15, 22, and 30, with a 71–100% probability of being estimated accurately (Figure 2B).

**Table 1:** Tau-U analysis of dysesthesia and upper limb activity parameters.

Phase effects								Weighted average				
		Baseline corrected									95% CI	
			S	Tau	VARs	SD	Z		Tau	Z	Lower	Upper
Tingling												
Pre	A1 vs. B1	No	−49	−1.00	245	15.65	−3.13**	Treatment effect	−0.92	−4.07**	−1.00	−0.48
	A2 vs. B2	No	−41	−0.84	245	15.65	−2.62**					
Post	A1 vs. B1	No	−49	−1.00	245	15.65	−3.13**	Treatment effect	−1.00	−4.43**	−1.00	−0.56
	A2 vs. B2	No	−49	−1.00	245	15.65	−3.13**					
Post1h	A1 vs. B1	no	−49	−1.00	245	15.65	−3.13**	Treatment effect	−1.00	−4.43**	−1.00	−0.56
	A2 vs. B2	no	−49	−1.00	245	15.65	−3.13**					
Allodynia												
Pre	A1 vs. B1	no	−49	−1.00	245	15.65	−3.13**	Treatment effect	−0.70	−3.12**	−1.00	−0.26
	A2 vs. B2	no	−20	−0.41	245	15.65	−1.28					
Post	A1 vs. B1	no	−49	−1.00	245	15.65	−3.13**	Treatment effect	−1.00	−4.43**	−1.00	−0.56
	A2 vs. B2	no	−49	−1.00	245	15.65	−3.13**					
Post1h	A1 vs. B1	no	−49	−1.00	245	15.65	−3.13**	Treatment effect	−1.00	−4.43**	−1.00	−0.56
	A2 vs. B2	no	−49	−1.00	245	15.65	−3.13**					
Mag ratio												
	A1 vs. B1	no	49	1.00	245	15.65	3.13**	Treatment effect	1.00	4.33**	0.55	1.00
	A2 vs. B2	no	35	1.00	152	12.32	2.84**					
Bi mag												
	A1 vs. B1	no	21	0.43	245	15.65	1.34	Treatment effect	0.42	1.81	−0.04	0.87
	A1 vs. A3	yes	7	0.20	152	12.32	0.57					

**p < 0.01.

Mag ratio, magnitude ratio; Bi mag, bilateral magnitude; CI, confidence interval; SD, standard deviation; VARs, Variances.

### 4.2 Effect of DM-TENS on allodynia

Supplementary Video 1 presents the immediate effect of DM-TENS for allodynia. Defensive pain response disappearance and reduced allodynia were observed. Figure 2C presents tingling at pre-treatment, post-treatment, and 1-h post-treatment. Tau-U analysis revealed significantly improved treatment effects at pre-treatment, post-treatment, and 1-h post-treatment (Table 1). The BUCP model revealed three change points for pre-treatment with a 53.6% probability, estimated to be on days 9, 19, and 30, with a 75.6–88.5% probability of being estimated accurately. Four change points were found for post-treatment with a 100% probability, estimated to be on days 8, 15, 22, and 29, with a 100% probability of being estimated accurately. For pre-treatment, four change points had a 96.8% probability, estimated to be on days 8, 15, 23, and 29, with a 99.4–100% probability of being estimated accurately (Figure 2D).

### 4.3 Effect of DM-TENS on upper limb activities

The mean scores of MAL were as follows: AOU: A1 = 2.33, B1 = 3.33, A2 = 3.00, B2 = 3.78, and A3 = 3.44 and QOM: A1 = 1.44, B1 = 2.67, A2 = 2.11, B2 = 3.44, and A3 = 2.89 (Supplementary Table 3).

**Figure 3:**
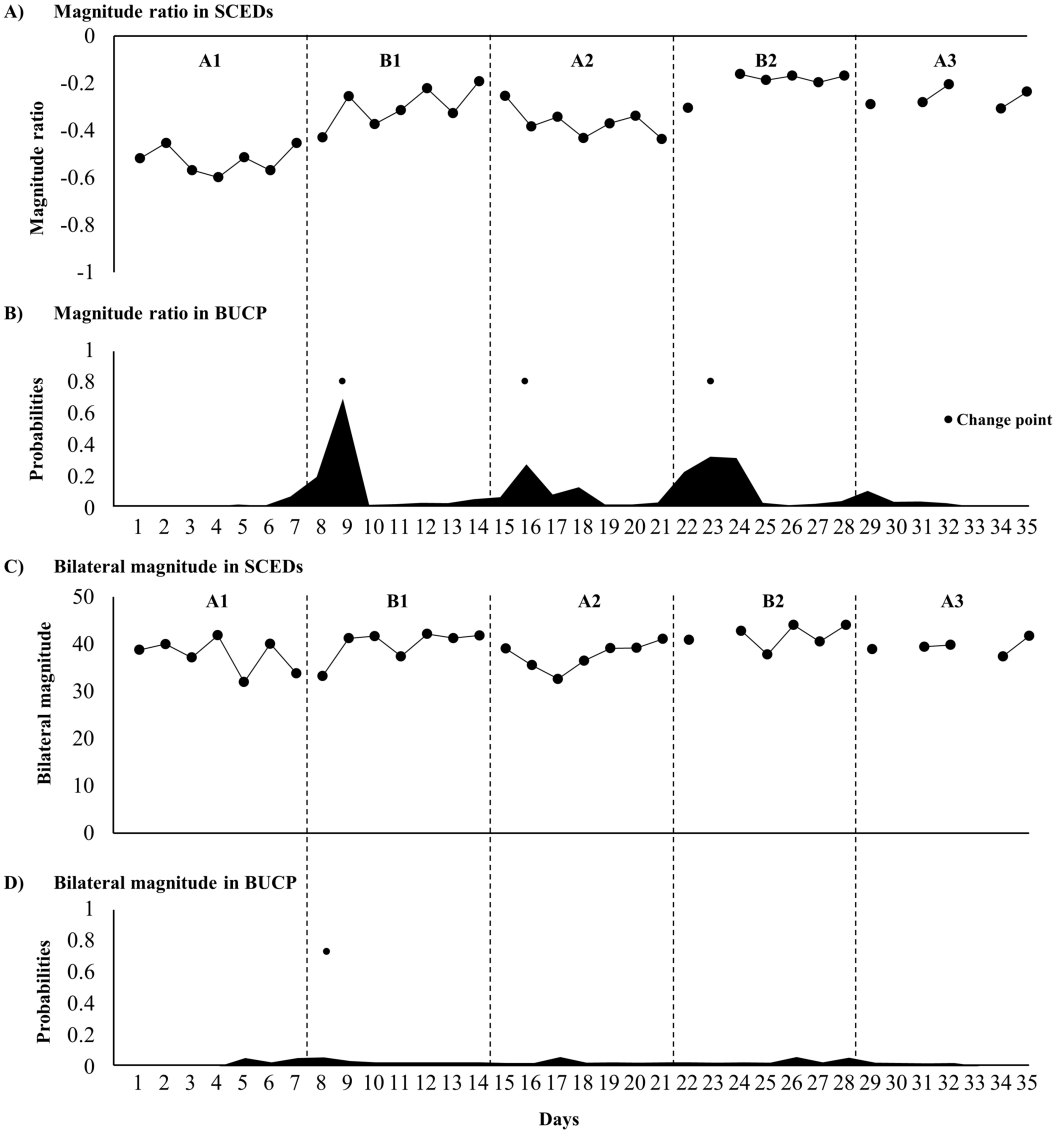
Time course of upper limb activity parameters and change point probabilities. **(A)** Time course of the magnitude ratio; **(B)** probabilities of magnitude ratio change points; **(C)** time course of the bilateral magnitude; and **(D)** probabilities of bilateral magnitude change points.

Wrist-worn accelerometers used to measure upper-limb activity were worn for <8 h on days 23, 30, and 33 (out of 35 days) (Figures 3A,C), which were excluded from the analysis. Tau-U analysis revealed a significantly improved magnitude ratio. The BUCP model revealed three change points for pre-treatment with a 41.5% probability, estimated to be on days 9, 16, and 23, with a 46.4–93.3% probability of being estimated accurately. However, the bilateral magnitude was not significantly different from that of the treatment phase (Figure 3B). One change point was found for post-treatment with a 44.1% probability, estimated to be on day 8, with a 14.4% probability of being estimated accurately (Figure 3D).

## 5 Discussion

We present the case of a patient with TM treated with DM-TENS following an intervention design. Tingling and allodynia were similarly improved following treatment; however, the carry-over effect was not observed for allodynia. Upper limb activity also improved. In the BUCP models, change-point estimates and their accuracy (probability) supported treatment effect immediacy and carry-over.

DM-TENS showed an immediate effect on tingling, decreasing from day 1 in the intervention phase (B1 and B2). After the intervention phase, the same effect was only maintained for 1–3 days; however, in phase A3, the carry-over effect occurred at approximately NRS 5. These results are consistent with our previous study, suggesting that DM-TENS has long-term effects on tingling due to central nervous system (CNS) diseases (Nishi et al., 2022). The primary analgesic mechanism of TENS involves peripheral blockade of nociceptive impulses, affecting the peripheral nervous system (gate control theory) and influencing the CNS through descending pain inhibition (Kasat et al., 2014; Melzack and Wall, 1965; Vance et al., 2014). These effects induce sensory disturbances and mechanical sensory reduction as side effects (Meyer-Frießem et al., 2019). However, DM-TENS exhibits a phenomenon where the sensations of tingling and electrical stimulation cancel each other, which is not observed with conventional TENS (Nishi et al., 2022). This phenomenon was also observed in the present study. We infer that DM-TENS acts in a mechanism different from that of conventional TENS. Electrical stimulation amplitude reflects the number of firing nerve fibers; the frequency affects neuron firing frequency (Deer et al., 2019). DM-TENS selectively blocks the sensory nerves specific to tingling with a tingling sensation in the peripheral nervous system (the “busy line” effect). This might lead to an immediate blockade, reducing tingling sensation and TENS input. Because the input to wide dynamic range spinal neurons and the sensory cortex is reduced, excessive excitation is suppressed, contributing to the sustained effect. DM-TENS was hypothesized to be ineffective against stabbing or cold stimulation in NPSI because of this tingling-specific mechanism.

Mechanical allodynia involves non-nociceptive stimuli perceived as pain through the Aβ fibers or low-threshold Aδ- and C-fibers. DM-TENS also immediately affected mechanical allodynia from day 1, decreasing it during the intervention phases (B1 and B2). The diurnal changes during the intervention phases showed the largest improvement during and immediately post-DM-TENS intervention, with the carry-over effects disappearing after approximately 5 h. After the intervention phase, the same effect was maintained for 1–5 days. The effects immediately and 1 h after the intervention were less likely to be retained.

One mechanism of allodynia is central sensitization, resulting from damage to central neurons; it reflects an increase in the excitability of neurons in the CNS, including the spinal cord (Finnerup et al., 2021). These changes result in a decrease in the activation threshold, increased activation, change in the distribution and spatial extent, and the recruitment of new inputs. The immediate effects of DM-TENS on allodynia were significant; however, the long-term effects lasted only 1–5 days, according to the BUCP model. The “busy line” effect of DM-TENS may selectively suppress the excitability of the spinal regions. However, suppressing the excitability of hyperinnervated regions may be impossible due to central sensitization, and long-term effects may be lacking. The patient’s TM was also not fully treated; TM-induced neurological symptoms persist, and the efficacy of DM-TENS for allodynia may not be sustained.

Regarding upper limb activity, the effects of DM-TENS improved the magnitude ratio but did not influence the bilateral magnitude, encompassing both limbs’ activity. We speculated that the patient compensated for the decrease in the affected upper limb’s activity due to sensory impairment by increasing the unaffected limb’s activity. The high TSK-11 score indicated that the affected limb’s activity was reduced due to dysesthesia-related kinesiophobia, possibly explained by the fear-avoidance model (Leeuw et al., 2007). Following dysesthesia improvement by DM-TENS, the affected upper limb’s activity increased, and compensatory behavior decreased. Despite the absence of long-term DM-TENS effect on allodynia, upper limb activity was maintained post-treatment. Kinesiophobia was reduced, showing that the patient could control dysesthesia with DM-TENS. It was highly significant that the recognition of subjective upper limb use improved alongside objective upper limb activity. This study has three key limitations. First, upper limb activity data was missing on days 23, 30, and 33. Second, while habituation effects were minimal, a slight adjustment in stimulation was needed in phase B1. Third, central sensitization, which can affect treatment outcomes, was not assessed (Arendt-Nielsen et al., 2018). As a result, the findings may not be fully generalizable to all patients undergoing TENS therapy, particularly those with different sensory thresholds or clinical conditions. Future research should incorporate measures of central sensitization to enhance the understanding of TENS efficacy in diverse cases.

## 6 Conclusion

Implementing DM-TENS in one patient with TM led to an immediate improvement in treatment-resistant tingling and allodynia; however, no long-term effect on allodynia was noted. However, a long-term effect on the affected upper limb’s activity was observed; the carry-over effect for tingling or the combination of DM-TENS may have influenced this effect. DM-TENS is expected to be effective even for rare and treatment-resistant TM dysesthesia.

## Data Availability

The original contributions presented in the study are included in the article/Supplementary material, further inquiries can be directed to the corresponding author.

## Footnotes

^1^http://www.Singlecaseresearch.org/calculators/tau-u
