# Supplementary material for: Case report: A novel transcutaneous electrical nerve stimulation improves dysesthesias and motor behaviors after transverse myelitis
Source: Front Hum Neurosci. 2024 Nov 6;18:1447029. doi: 10.3389/fnhum.2024.1447029 (PMC11609914; doi:10.3389/fnhum.2024.1447029)
Supplement: Supplementary file 1 [file Data_Sheet_1.docx]

Supplementary Material

# Supplementary Figure 1





T2-weighted images of the cervical spinal cord. (A) Sagittal plane images before and (B) after extended laminectomy and steroid therapy. (C) Axial plane images before and (D) after extended laminectomy and steroid therapy.

# Supplementary Figure 2

#



Placement of transcutaneous electrical nerve stimulation (TENS) electrodes.The distance between the centers of TENS electrodes was 6 cm.

# Supplementary Table 1

|  | DTR | | MMT | | Light touch | | Pin prick | |
| --- | --- | --- | --- | --- | --- | --- | --- | --- |
|  | Right | Left | Right | Left | Right | Left | Right | Left |
| C2 | N/A | N/A | N/A | N/A | 2 | 2 | 2 | 2 |
| C3 | N/A | N/A | N/A | N/A | 2 | 2 | 2 | 2 |
| C4 | N/A | N/A | N/A | N/A | 2 | 2 | 2 | 2 |
| C5 | 2+ | 3+ | 5 | 5 | 2 | 2 | 2 | 2 |
| C6 | 2+ | 3+ | 5 | 5 | 2 | 2 | 2 | 2 |
| C7 | 2+ | 3+ | 5 | 4 | 2 | 2 | 2 | 2 |
| C8 | N/A | N/A | 5 | 4 | 1 | 1 | 1 | 1 |
| T1 | N/A | N/A | 5 | 4 | 1 | 1 | 1 | 1 |
| T2 | N/A | N/A | N/A | N/A | 1 | 1 | 1 | 1 |
| T3 | N/A | N/A | N/A | N/A | 1 | 1 | 1 | 1 |
| T4 | N/A | N/A | N/A | N/A | 1 | 1 | 1 | 1 |
| T5 | N/A | N/A | N/A | N/A | 1 | 1 | 1 | 1 |
| T6 | N/A | N/A | N/A | N/A | 1 | 1 | 1 | 1 |
| T7 | N/A | N/A | N/A | N/A | 1 | 1 | 1 | 1 |
| T8 | N/A | N/A | N/A | N/A | 1 | 1 | 1 | 1 |
| T9 | N/A | N/A | N/A | N/A | 1 | 1 | 1 | 1 |
| T10 | N/A | N/A | N/A | N/A | 1 | 1 | 1 | 1 |
| T11 | N/A | N/A | N/A | N/A | 1 | 1 | 1 | 1 |
| T12 | N/A | N/A | N/A | N/A | 1 | 1 | 1 | 1 |
| L1 | N/A | N/A | N/A | N/A | 1 | 1 | 1 | 1 |
| L2 | N/A | N/A | 3 | 3 | 1 | 1 | 1 | 1 |
| L3 | N/A | N/A | 4 | 4 | 1 | 1 | 1 | 1 |
| L4 | 3+ | 3+ | 4 | 4 | 1 | 1 | 1 | 1 |
| L5 | N/A | N/A | 4 | 3 | 1 | 1 | 1 | 1 |
| S1 | 3+ | 3+ | 3 | 3 | 1 | 1 | 1 | 1 |
| S2 | N/A | N/A | N/A | N/A | 1 | 1 | 1 | 1 |
| S3 | N/A | N/A | N/A | N/A | 1 | 1 | 1 | 1 |
| S4-5 | N/A | N/A | N/A | N/A | 1 | 1 | 1 | 1 |
| Total | N/A | N/A | 43 | 39 | 34 | 34 | 34 | 34 |

[footnote] Neurological Examination of each spinal cord segment. DTR was assessed using the British scale. Motor and sensory examinations were assessed using the International Standards for Neurological Classification of spinal cord injury. DTR: Deep tendon reflex, MMT: Manual muscle testing, N/A: not available

# Supplementary Table 2

|  | A1 | B1 | A2 | B2 | A3 |
| --- | --- | --- | --- | --- | --- |
| Burning | 3 | 2 | 3 | 2 | 2 |
| Squeezing | 0 | 0 | 0 | 0 | 0 |
| Pressure | 0 | 0 | 0 | 0 | 0 |
| Duration of spontaneous pain | over 12h | over 12h | over 12h | over 12h | over 12h |
| Electric shock | 10 | 4 | 9 | 4 | 6 |
| Stabbing | 10 | 8 | 10 | 8 | 10 |
| Number of pain attacks | 11 to 20 | 6 to 10 | 11 to 20 | 6 to 10 | 11 to 20 |
| Provoked by brushing | 10 | 4 | 10 | 3 | 8 |
| Provoked by pressure | 8 | 4 | 8 | 4 | 6 |
| Provoked by cold stimulation | 9 | 5 | 9 | 5 | 9 |
| Pins and needles | 9 | 4 | 8 | 3 | 4 |
| Tingling | 10 | 4 | 8 | 3 | 5 |
| Total score | 69 | 35 | 65 | 32 | 50 |

[Footnote] Neuropathic pain symptom inventory (NPSI) in each phase. The temporal items assessing the duration of spontaneous ongoing pain and the number of pain attacks were not analyzed in this study.

# Supplementary Table 3

|  | AOU | | | | |  | QOM | | | | |
| --- | --- | --- | --- | --- | --- | --- | --- | --- | --- | --- | --- |
|  | A1 | B1 | A2 | B2 | A3 |  | A1 | B1 | A2 | B2 | A3 |
| Hold book | 2 | 3 | 3 | 4 | 4 |  | 1 | 3 | 3 | 4 | 3 |
| Use towel | 2 | 3 | 3 | 3 | 3 |  | 2 | 2 | 2 | 3 | 2 |
| Pick up glass | 5 | 5 | 5 | 5 | 5 |  | 2 | 3 | 3 | 4 | 4 |
| Brush teeth | 0 | 3 | 1 | 4 | 2 |  | 0 | 3 | 1 | 4 | 3 |
| Shave/Make-up | － | － | － | － | － |  | － | － | － | － | － |
| Open door with key | － | － | － | － | － |  | － | － | － | － | － |
| Write/Type | 1 | 3 | 2 | 4 | 3 |  | 1 | 3 | 2 | 4 | 3 |
| Steady self | 4 | 4 | 4 | 4 | 4 |  | 1 | 2 | 1 | 2 | 2 |
| Put arm through clothing | 4 | 4 | 4 | 4 | 4 |  | 4 | 4 | 4 | 4 | 4 |
| Carry object | 2 | 3 | 3 | 3 | 3 |  | 1 | 2 | 1 | 3 | 2 |
| Grasp fork/spoon | － | － | － | － | － |  | － | － | － | － | － |
| Comb hair | － | － | － | － | － |  | － | － | － | － | － |
| Pick up cup | － | － | － | － | － |  | － | － | － | － | － |
| Button clothes | 1 | 2 | 2 | 3 | 3 |  | 1 | 2 | 2 | 3 | 3 |
| Mean score | 2.33 | 3.33 | 3.00 | 3.78 | 3.44 |  | 1.44 | 2.67 | 2.11 | 3.44 | 2.89 |

[Footnote] The motor activity log (MAL) in each phase. The mean of amount of use (AOU) and quality of movement (QOM) were calculated after excluding items that were performed only with the unaffected hand before transverse myelitis (TM).

# Supplementary Table 4

| Description | A1 | B1 | A2 | B2 | A3 |
| --- | --- | --- | --- | --- | --- |
| I'm afraid that I might injure myself if I exercise | 3 | 1 | 2 | 1 | 1 |
| If I were to overcome it, my pain would increase | 3 | 2 | 2 | 2 | 2 |
| My body is telling me I have something dangerously wrong | 3 | 3 | 3 | 2 | 3 |
| People aren't taking my medical condition seriously enough | 2 | 2 | 2 | 2 | 2 |
| My accident has put my body at risk for the rest of my life | 3 | 3 | 3 | 3 | 3 |
| Pain always means I have injured my body | 3 | 3 | 3 | 3 | 3 |
| Simply being careful that I do not make any unnecessary movements is the safest thing I can do to prevent my pain from worsening | 2 | 1 | 2 | 1 | 2 |
| I wouldn't have this much pain if there wasn't something potentially dangerous going on in my body | 2 | 2 | 2 | 2 | 2 |
| Pain lets me know when to stop exercising so that I don't injure myself | 2 | 2 | 2 | 2 | 2 |
| I can't do all the things normal people do because it's too easy for me to get injured | 3 | 2 | 3 | 2 | 2 |
| No one should have to exercise when he/she is in pain | 2 | 2 | 2 | 2 | 2 |
| Total score | 28 | 23 | 26 | 22 | 24 |

[Footnote] TSK-11 in each phase.

# Supplementary Video 1

The immediacy dysesthesia-matched transcutaneous electrical nerve stimulation (DM-TENS) effect for allodynia. DM-TENS was conducted for 1 min in this movie.
